# The development and validation of a resource consumption score of an emergency department consultation

**DOI:** 10.1371/journal.pone.0247244

**Published:** 2021-02-19

**Authors:** Martin Müller, Clyde B. Schechter, Wolf E. Hautz, Thomas C. Sauter, Aristomenis K. Exadaktylos, Stephanie Stock, Tanja Birrenbach

**Affiliations:** 1 Department of Emergency Medicine, Inselspital, University Hospital, University of Bern, Bern, Switzerland; 2 Institute of Health Economics and Clinical Epidemiology, University Hospital of Cologne, Cologne, Germany; 3 Department of Family & Social Medicine & Department of Epidemiology Population Health, Albert Einstein College of Medicine, Bronx, New York, United States of America; 4 Center for Educational Measurement, University of Oslo, Oslo, Norway; Sunnybrook Research Institute, CANADA

## Abstract

**Background:**

Emergency Department (ED) visits and health care costs are increasing globally, but little is known about contributing factors of ED resource consumption. This study aims to analyse and to predict the total ED resource consumption out of the patient and consultation characteristics in order to execute performance analysis and evaluate quality improvements.

**Methods:**

Characteristics of ED visits of a large Swiss university hospital were summarized according to acute patient condition factors (e.g. chief complaint, resuscitation bay use, vital parameter deviations), chronic patient conditions (e.g. age, comorbidities, drug intake), and contextual factors (e.g. night-time admission). Univariable and multivariable linear regression analyses were conducted with the total ED resource consumption as the dependent variable.

**Results:**

In total, 164,729 visits were included in the analysis. Physician resources accounted for the largest proportion (54.8%), followed by radiology (19.2%), and laboratory work-up (16.2%). In the multivariable final model, chief complaint had the highest impact on the total ED resource consumption, followed by resuscitation bay use and admission by ambulance. The impact of age group was small. The multivariable final model was validated (R^2^ of 0.54) and a scoring system was derived out of the predictors.

**Conclusions:**

More than half of the variation in total ED resource consumption can be predicted by our suggested model in the internal validation, but further studies are needed for external validation. The score developed can be used to calculate benchmarks of an ED and provides leaders in emergency care with a tool that allows them to evaluate resource decisions and to estimate effects of organizational changes.

## Introduction

Increasing healthcare costs are a worldwide problem [1]. A substantial proportion of these costs results from Emergency Departments (ED), as these provide nearly half of the hospital-associated medical care nowadays in Western countries [2]. ED visits are rising globally [3], and over and above, ED care is more expensive compared to other forms of healthcare [4]. Furthermore, in times of a global healthcare and economic crisis, as in the on-going COVID-19 pandemic, an efficient allocation of material and human resources in the ED is crucial to ensure medical care that is economically sustainable [5].

Despite the important role of ED care in the healthcare system, ED resource consumption has only been modestly studied so far [6–15]. Furthermore, instead of reporting the actual resource consumption, some studies rely on surrogate measures to represent resource consumption, i.e. arrival by ambulance, triage category, number of tests and procedures performed, length of stay in the ED, or admission rates [7, 14]. Most studies reported a positive association of increasing age [8–10, 12, 14] and higher acuity triage category [6, 7, 10, 11, 16] with resource consumption. The evaluation of EDs in terms of resource and performance analysis was often based solely on the volume of an ED counting, for example the number of patients treated each year [17]. However, the profile of the patients admitted to a medical department often varies considerably–consequently so do the resources required by each ED [7]. Thus, assessments of efficiency must take the treated patient profile into consideration.

Within intensive care units, an adapted version of the “Therapeutic Intervention Scoring System” (TISS) [18] is often used as a tool to perform resource and performance analysis [19, 20]. The average overall TISS-28 score per patient day or nurse, for instance, is used to measure the performance of intensive care units in Switzerland and internationally [21–24]. To our knowledge an analogue score that evaluates the use of resources in ED departments does not yet exist. Such a score has the potential to identify areas and special ED patient groups with high resource demands at an early stage–a prerequisite to implement preventive procedures and adaptive actions that might increase the structural, process, and performance quality in the ED in combination with optimisation of needed resources. Furthermore, it might be of great use in research as a standardised tool to describe the performance of an ED and to compare different EDs on an international level.

The aims of this study are i) to illustrate the distribution of an ED patient’s needed resources in different subgroups (laboratory, nurse, physician, material, and radiology), ii) to identify factors that are associated with the total ED resources, iii) to develop and validate a scoring system that predicts the ED resource consumption of a patient and demonstrate a practical example of application for quality assurance.

## Methods

### Study design, site and period

This is a retrospective cohort analysis of all adult patients admitted to the ED at Inselspital, University Hospital, University of Bern). The ED of Inselspital is one of the largest EDs in Switzerland with a catchment area of two million people, and about 50,000 ED consultations per year [25]. The study period is over five years from 01.01.2013 to 31.12.2017. There were no major structural changes during the study period (see S1 Appendix for a detailed description of our ED and patient management process).

### Eligibility criteria

All adult patients (age≥18) presenting to the ED over the study period were included. Patients were excluded if i) the case identification number or a documented chief complaint was missing, ii) multiple consultations shared one case identification number (e.g. very short-term revisits), iii) the consultation generated few or no entries (total ED resources less than 10 tax points, see below) in the resource databases (e.g. cancellations, incomplete documentation), and iv) patients were seen by the psychiatrist as the leading ED physician, as they have a different billing system.

### ED resource consumption

Every procedure that is performed in the ED is documented by the person who performed the procedure with a procedural code out of the *TARMED Suisse catalogue* [26] given by the Swiss health law for billing purposes. Although not all procedural codes are billing-relevant, those codes form the basis for the billing. All members of the ED are trained regularly to achieve accurate coding.

Two-hundred fifty-nine specific procedural codes of the *TARMED Suisse catalogue* [26] that are regularly used in our ED were chosen by a working group consisting of acute care nurses, radiology nurses, ED physicians, and the controller of our ED department. A numeric value is assigned for each procedural code (e.g. *00*.*0410* brief physical examination of a patient) with corresponding unit/”medical currency” *tax points*. The medical currency one tax point (TP) roughly corresponds to 1 US-$, but the exact amount varies among hospitals.

Those codes were grouped into different resource groups i.e. physician, nurse, laboratory, radiology, and material, see S2 Appendix). *The total ED resource consumption of a consultation* was defined as the sum of all TP of all defined codes. Additionally, the total ED costs for each patient were obtained.

As a secondary outcome and additional surrogate marker of ED resource consumption, the length of stay (LOS) in the ED was also extracted.

### Potential predictor variables

Contextual factors and factors describing the acute as well as the chronic condition of a patient’s consultation were assessed as potential predictor variables:

1. *Contextual factors*: season of the year (spring to winter), Saturday or Sunday admission, and night-time admissions (from 19:00 to 06:59), the occupancy index (defined as the ratio between the total number of patients in the ED and the total number of ED treatment beds) [27, 28] and the emergency department work index (EDWIN) calculated as (Σ *n*_*i*_ x *t*_*i*_) / [*N*_*a*_ x (*B*_*T*_—*B*_*A*_)], where *n*_*i*_ = number of patients in the ED in triage category *i*, *t*_*i*_ = triage category, *N*_*a*_ = number of attending physicians on duty, *B*_*T*_ = the number of ED treatment beds, *B*_*A*_ = total number of admitted patients in the ED (0–1.5, active ED; 1.5–2.0, very busy ED; >2, overcrowded ED) [29, 30]. The two latter factors were used to describe the business of the ED.
2. *Acute condition*: type of admission, chief complaint groups such as trauma and neurological complaint (see S4 Appendix, based on Aronsky et al. [31]), and documented vital deviations i.e. oxygen saturation (<90%), systolic blood pressure (<100mmHg), temperature (<35.0°C or >38.5°C), level of consciousness (Glascow Coma Scale <15), respiratory rate (<8/min or >25/min), and heart rate (<50/min or >110/min) as these deviations are associated with severe disease courses and higher mortality [32]. To reflect polytrauma and unstable patients, the need for resuscitation room care was defined as a potential predictor variable.
  For sensitivity analysis, instead of vital deviations, the triage group was used, which is routinely assessed by special trained nurses using the Swiss Emergency Triage Scale [33], a triage scale similar to the Manchester Triage System [34] (1: highly acute to 5: non-urgent).
3. Chronic conditions:
  - Important comorbidities based on the Charlson Comorbidity Index [35]: COPD, diabetes, liver disease, dementia, malignancy, cerebrovascular disease, peripheral artery disease, coronary vessel disease, and chronic kidney disease.
  - Drug intake (on admission or discharge) based on the Anatomical Therapeutic Chemical (ATC) classification system [36]: antidiabetic (ATC code A10), antithrombotic (B01), antihypertensive (C02, C04-C09), diuretic (C03), opioid (N02A), and–to set neurological patients, who are thought to have high ED resource consumption, in a broader context–antiepileptic (N03) and psycholeptic (N05).
4. *Other*: Demographic factors: age and sex.

### Data extraction

The potential predictor variables were extracted from the computerized clinical databases (E-Care, ED 2.1.3.0, Turnhout, Belgium). All procedural codes were extracted from the administrative database (OpenText Suite for SAP® Solutions, OpenText Corporation, Waterloo, Canada). For a detailed protocol, including the definitions of the variables and validation, see S2–S4 Appendices.

### Ethical considerations

The study was performed in accordance with Swiss law. The Bern ethics committee registered the study as a quality assurance study (2018–00198) and waived the requirement for informed consent.

### Statistical analysis

Stata® 13.1 (StataCorp, The College Station, Texas, USA) was used for statistical analysis. All continuous variables are presented as medians with 25^th^- 75^th^ percentile ranges (IQR). Categorical variables are shown with frequency and proportion. The outcome was natural logarithm (ln)-transformed account for the skewness of the total ED resource consumption. Univariable linear regression analysis with the transformed outcome was performed to quantify the association of the total ED consumption and potential score parameters. The exponentiated coefficient of such a model correspond to the geometric mean ratio (GMR) of the non-log-transformed outcome in the presence vs. the absence of the predictor [37].

For the score development the dataset was randomly split (50:50) into a training and validation set. All studied predictor variables were included in a multivariable linear regression analysis with the ln-transformed total ED resource consumption as outcome. For a parsimonious final model, predictors that changed the geometric mean by less than 10% (0.9 < GMR < 1.1) were removed stepwise from the final model.

The total ED resource consumption can be predicted from the linear regression model as

$$\mathrm{Total}\;\mathrm{ED}\;\mathrm{resources}=\exp\left(a_{0}+\sum_{i=1}^{m}a_{i}\times x_{i}\right),$$

Where *a*_0_ is the constant and *a*_1_,…,*a*_*m*_ are the coefficients of the final model and *x*_1_,…,*x*_*m*_ are binary variables (0/1) indicating the presence or absence of the predictor. From the final model, the resource score, will be defined as

$$\mathrm{Total}\;\mathrm{ED}\;\mathrm{resource}\;\mathrm{score}=c_{0}\times \left(c_{1+}+a_{0}+\sum_{i=1}^{m}a_{i}\times x_{i}\right),$$

where the constants *c*_0_ and *c*_1_ are determined, so that the obtained score possibly ranges from 0 to 100.

Different sensitivity analyses for the final model were performed: A model i) with the use of the triage category instead of the vital parameters (model 2), ii) with an additional interaction term between trauma and resuscitation room use to better reflect polytrauma (model 3), and iii) excluding revisits (model 4). One might argue that the Swiss *Tarmed* codes do not validly reflect resource consumption as a “resource measure” is already assigned to each process. Thus, we also evaluated the parameter LOS in the ED (in hours) as an outcome, applying the same model development procedure.

To assess the fit and parsimony of the ED resource consumption models, predictive accuracy, and explained variance, the following parameters were calculated: Akaike information criterion (AIC), and Bayesian information criterion (BIC) for the development sample, and mean absolute prediction error (MAPE), mean relative squared error (MRSE), mean squared prediction error (MSPE) and R^2^ for the validation sample [38]. The obtained R^2^ was compared to a model that included triage and age group as predictor variables only. Furthermore, for a more intuitive measure of predictive accuracy, we calculated the median and IQR of the absolute percentage of the deviation of the predicted to observed ratio (|100%—predicted/observed x 100%|) for all deciles in the different models [39].

## Results

### Patients’ demographics

In total, 164,729 out of 206,006 consultations were included in the analysis and were randomised 1:1 into validation (n = 82,341) and training sets (n = 82,388). The reasons for exclusion were i) patient age younger than 18 years (n = 6,992), ii) case identification number missing or associated with multiple consultations (n = 7,478), iii) few or no (<10 TP) resource database entries, e.g. cancelled consultation (n = 7,639), iv) the psychiatrist was the leading physician (n = 8,511), or v) the chief complaint was not documented (n = 10,657), see S5 Appendix.

In the study population, the median age was 49 years (IQR 32, 67), with 56.3% males. The most common triage group was urgent (60.7%). In total, 16.3% of the consultations had a neurological chief complaint and 16.7% of the patients presented after trauma, and 35.2% of the ED consultations led to hospitalization. There were no significant differences between the validation and training set (S6 Appendix).

#### Distribution of ED resource consumption

The median total ED resource consumption was 638 (IQR 254, 1264) TP in the training set. The median in the resource subgroup was highest for the physician resources (training set: 323 TP, IQR 119, 500) followed by laboratory work-up (training set: 96.4 TP, IQR 0, 227) and radiological work-up (training set: 60 TP, IQR 0, 420) with no difference between the training and validation set (S7 Appendix).

The mean relative distribution was slightly different: Physician resources made up the largest proportion (54.8%), followed by radiology (19.2%), laboratory work-up (16.2%), nursing resources (5.4%), and materials (4.5%) in the training set.

**Fig 1:**
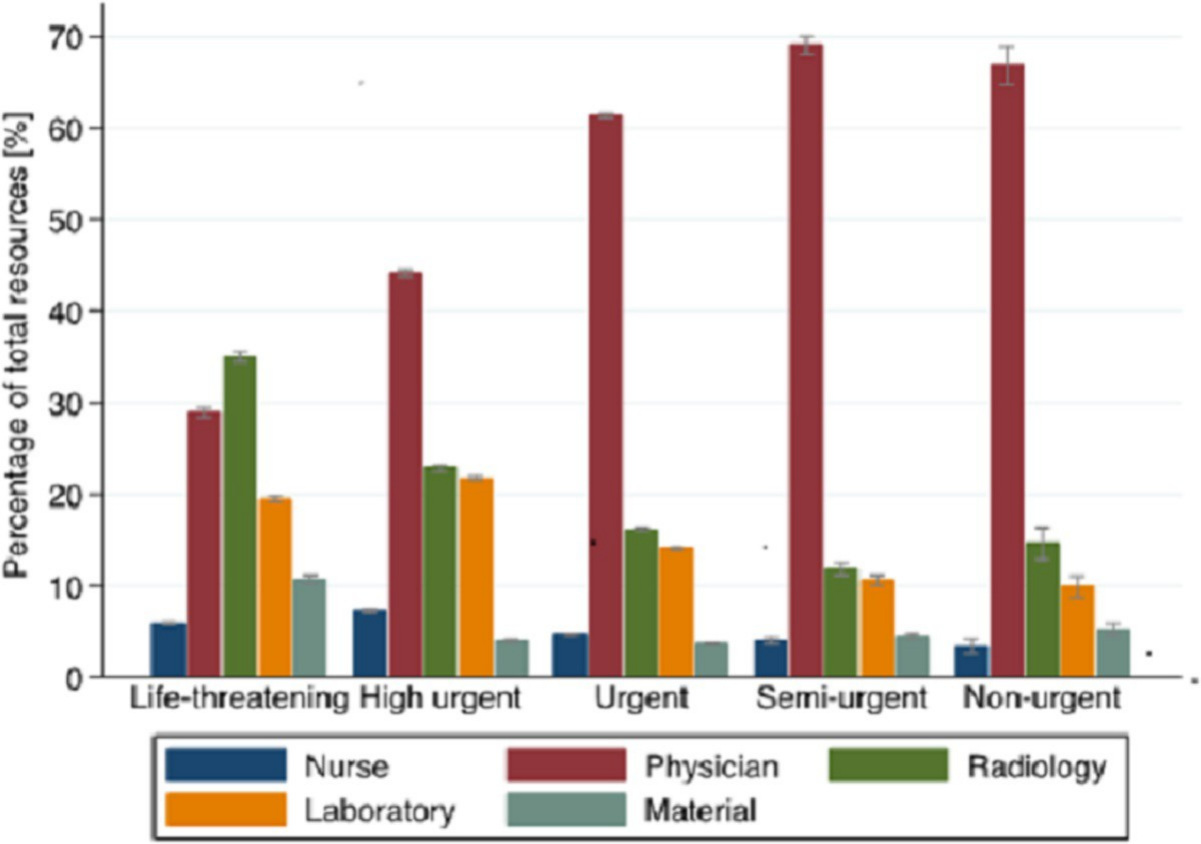
Comparison of the distribution of the total ED resource consumption (mean percentage of total resources and 95% CI bars) by triage category (n = 82,388*). *the triage category was missing in 1,148 consultations.

The distribution of the total ED resource consumption by triage category is shown in Fig 1. Physician resources accounted for the major percentage in all but the life-threatening triage category. The proportions of radiology and laboratory resources were higher in the life-threatening and high urgent categories than in the less acute triage groups.

The correlation of ln-transformed total ED resource consumption and total ED costs was high; Pearson’s correlation coefficient was 0.939 (95% CI: 0.939–0.940).

### Prediction of total ED resource consumption

Table 1 shows the univariable associations of the acute patient condition factors with the total ED resource consumption. Ambulance admission and resuscitation bay use increased the geometric mean about the factor 2.5 (2.7 and 2.5). Compared to urgent triage, semi-urgent triage had less (GMR 0.5) while high urgent (GMR 2.3) and life-threatening (GMR 4.6) had more resource needs. Last, the chief complaint group had a high impact on resource consumption ranging from a geometric mean factor 0.2 (eye problem) to 2.9 (neurological complain) compared to resources of patients presenting with musculoskeletal complains.

**Table 1:** Univariable association of acute patient condition factors with the total ED resource consumption (n = 164,729).

	GMR	95% CI	p-value
**Type of admission**			
Ambulance admission	2.67	(2.63, 2.71)	<0.001
**Chief complaint group**			
Cardiovascular	2.14	(2.10, 2.19)	<0.001
Ear/Nose/Throat	0.28	(0.27, 0.29)	<0.001
Eye	0.16	(0.16, 0.17)	<0.001
Gastrointestinal	1.55	(1.52, 1.58)	<0.001
Genitourinary	1.03	(1.00, 1.06)	0.021
Musculoskeletal	1.00	(base)	
Neurological	2.88	(2.83, 2.94)	<0.001
Respiratory	2.00	(1.95, 2.05)	<0.001
Trauma	1.37	(1.34, 1.40)	<0.001
Other	1.18	(1.16, 1.20)	<0.001
**Resuscitation bay use**	2.53	(2.49, 2.57)	<0.001
**Vital deviations**			
Heart rate (<50/min or >110/min)	1.49	(1.41, 1.59)	<0.001
Level of consciousness (GCS <15)	2.04	(2.00, 2.08)	<0.001
Oxygen saturation (<90%)	1.92	(1.83, 2.01)	<0.001
Respiratory rate (<8/min or >25/min)	2.25	(2.18, 2.32)	<0.001
Systolic blood pressure (<90mmHg)	1.76	(1.71, 1.82)	<0.001
Temperature (<35.0°C or >38.5°C)	3.01	(2.76, 3.29)	<0.001
**Triage**			
Life-threatening	4.56	(4.48, 4.65)	<0.001
High urgent	2.34	(2.31, 2.37)	<0.001
Urgent	1.00	(base)	
Semi-urgent	0.50	(0.49, 0.51)	<0.001
Non-urgent	0.96	(0.90, 1.02)	0.193

**Abbreviations:** CI, Confidence interval; GCS, Glascow Coma Scale; GMR, Geometric mean ratio.

Table 2 shows the univariable associations of chronic patient condition and contextual factors with the total ED resource consumption. The resource needs increased with increasing age, with the least resource consumption by 18–24 year olds, and the most for patients older than 85 years. The analysed drug intake and comorbidities increased the geometric mean by factors from 1.7 to 3.0. Apart from weekend admissions (GMR 0.8), the impact of contextual factors on ED resource consumption was small.

**Table 2:** Univariable association of chronic patient condition and contextual factors with the total ED resource consumption (n = 164,729).

	GMR	95% CI	p-value
**Sociodemographic characteristics**			
Age group, per year			
18–24	0.68	(0.67, 0.69)	<0.001
25–44	0.73	(0.72, 0.74)	<0.001
45–64	1.00	(base)	
65–84	1.34	(1.32, 1.36)	<0.001
≥85	1.57	(1.52, 1.61)	<0.001
Sex, male	0.96	(0.95, 0.97)	<0.001
**Comorbidities**			
Cerebrovascular disease	2.99	(2.93, 3.05)	<0.001
Chronic kidney disease	1.88	(1.82, 1.96)	<0.001
COPD	2.01	(1.94, 2.08)	<0.001
Coronary artery disease	2.08	(2.04, 2.12)	<0.001
Dementia	2.18	(2.09, 2.27)	<0.001
Diabetes	1.92	(1.88, 1.96)	<0.001
Liver disease	1.86	(1.80, 1.92)	<0.001
Malignancy	1.84	(1.80, 1.87)	<0.001
Peripheral artery disease	1.93	(1.86, 2.01)	<0.001
**Drug intake**			
On any antidiabetic	1.95	(1.90, 1.99)	<0.001
On any antiepileptic	1.89	(1.84, 1.93)	<0.001
On any antihypertensive	2.27	(2.24, 2.30)	<0.001
On any antithrombotic	2.26	(2.24, 2.29)	<0.001
On any diuretic	2.17	(2.13, 2.22)	<0.001
On any opioids	1.68	(1.64, 1.71)	<0.001
On any psycholeptic	1.89	(1.86, 1.92)	<0.001
**Contextual factors**			
Season of the year			
Winter	1.00	(base)	
Spring	0.98	(0.97, 1.00)	0.031
Summer	0.99	(0.98, 1.01)	0.459
Fall	1.03	(1.02, 1.05)	<0.001
Night-time admissions	1.02	(1.01, 1.03)	0.003
Saturday or Sunday admission (00:00–23:59)	0.81	(0.80, 0.82)	<0.001
Occupancy index, per % increase	1.00	(1.00, 1.00)	<0.001
EDWIN score			
0–1.5, active	1.00	(baseline)	
1.5–2.0, very busy	1.02	(0.99, 1.05)	0.143
>2, overcrowded	1.02	(0.89, 1.19)	0.742

**Abbreviations:** CI, Confidence interval; COPD, Chronic obstructive pulmonary disease; GMR, Geometric mean ratio.

The two analysed factors describing busyness of the ED, occupancy index and EDWIN score, did only slightly change the GMR (1.0, respectively 1.02).

### Development of a scoring system

The results of the multivariable linear regression analysis of the final model (see statistical analysis) are shown in Table 3. The highest impact on the total ED resource consumption had chief complaint with a GMR ranging from 0.2 (eye problems) to 2.3 (neurological complaints) compared to patients with musculoskeletal complaints (baseline group). Each documented vital parameter deviation (heart rate, blood pressure, oxygen saturation, respiratory rate, level of consciousness and temperature) increased the geometric mean of the total ED resource consumption between 11 and 23%. Resuscitation bay use increased the geometric mean by a factor of 2.3 and admission by ambulance by a factor of 1.4. Additionally, drug intake (antithrombotic, antihypertensive, and opioids) and comorbidities (liver disease, malignancy, and cerebrovascular disease) increased the geometric mean each about 18–19% and 25–32%. The impact of age on total ED resource consumption was smaller in the multivariable model than in the univariable analysis.

**Table 3:** Multivariable analysis to predict total ED resource consumption (ln-transformed) in the training set. The exponentiated coefficients (Coef.) correspond to the GMR.

	GMR	95% CI	p-value	Coef.*	Name
**Type of admission**					
Ambulance admission	1.44	(1.41, 1.46)	<0.001	3.6	a_1_
**Chief complaint group**					
Cardiovascular	1.65	(1.60, 1.70)	<0.001	5	a_2_
Ear/Nose/Throat	0.31	(0.30, 0.32)	<0.001	-11.7	a_3_
Eye problem	0.20	(0.19, 0.20)	<0.001	-16.3	a_4_
Gastrointestinal	1.51	(1.47, 1.56)	<0.001	4.1	a_5_
Genitourinary	1.09	(1.06, 1.13)	<0.001	0.9	a_6_
Musculoskeletal	1.00	(base)	<0.001	0	a_7_
Neurological	2.30	(2.24, 2.36)	<0.001	8.3	a_8_
Respiratory	1.54	(1.49, 1.60)	<0.001	4.3	a_9_
Trauma	1.25	(1.22, 1.28)	<0.001	2.3	a_10_
Other	1.09	(1.07, 1.12)	<0.001	0.9	a_11_
**Age group, per year**					
18–24	0.86	(0.85, 0.88)	<0.001	-1.5	a_12_
25–44	0.91	(0.90, 0.92)	<0.001	-1	a_13_
45–64	1.00	(base)	<0.001	0	a_14_
65–84	0.98	(0.97, 1.00)	0.026	-0.2	a_15_
≥85	0.97	(0.95, 1.00)	0.048	-0.3	a_16_
**Acuity**					
Blood pressure (systolic <100mmHg)	1.11	(1.08, 1.15)	<0.001	1.1	a_17_
Heart rate (<50/min or >110/min)	1.16	(1.10, 1.23)	<0.001	1.5	a_18_
Level of consciousness (GCS <15)	1.16	(1.14, 1.18)	<0.001	1.5	a_19_
Oxygen saturation (SpO2 < 90%)	1.11	(1.06, 1.16)	<0.001	1.1	a_20_
Respiratory rate (<8/min or >25/min)	1.23	(1.19, 1.26)	<0.001	2	a_21_
Resuscitation bay	2.34	(2.29, 2.40)	<0.001	8.5	a_22_
Temperature (<35.0°C or >38.5°C)	1.14	(1.05, 1.24)	0.002	1.3	a_23_
**Drug intake**					
On any antihypertensive	1.18	(1.16, 1.20)	<0.001	1.6	a_24_
On any antithrombotic	1.18	(1.16, 1.19)	<0.001	1.6	a_25_
On any opioids	1.19	(1.17, 1.22)	<0.001	1.8	a_26_
**Comorbidity**					
Cerebrovascular disease	1.27	(1.24, 1.30)	<0.001	2.4	a_27_
Liver disease	1.32	(1.28, 1.36)	<0.001	2.8	a_28_
Malignancy	1.25	(1.23, 1.28)	<0.001	2.3	a_29_

**Abbreviations:** CI, Confidence interval; Coef.; coefficient; GMR, Geometric mean ratio.

* Coefficients a_i_ of the linear regression model (for better reading multiplicated by the factor 10).

With the formula presented in the statistical analysis the section and the values for the coefficients presented in Table 3, for instance, the total ED resource consumption of a 30 year old patient with respiratory symptoms, normal vitals, without any comorbidity, and admitted by the ambulance are estimated to be

$$\mathrm{Total}\;\mathrm{ED}\;\mathrm{resources}=\text{exp}\left(6.0+\frac{1}{10}\left[-1+4.3+3.6\right]\right)=\text{exp}\left(6.69\right)\approx 804\text{TP}.$$

Out of the coefficients presented in Table 3, the total ED resource consumption score was thus defined as

$$\mathrm{Total}\;\mathrm{ED}\;\mathrm{resource}\;\mathrm{score}=1.7\times \left(17.8+\sum_{i=1}^{29}a_{i}\times x_{i}\right).$$

#### Validation of the scoring system and sensitivity analysis

The median resource score was 35.9 (IQR 31.5, 44.2) with a range of 0 to 84.3 in the validation set. The ED resource score was validated by validation of the multivariable model. Table 4 and S8 Appendix show the validation of the final model (model 1), as well as–for sensitivity analysis–the three other models. All sensitivity analysis models only slightly changed the observed R^2^ of 0.54, which means that 54% of the variance in the total ED resources (ln-transformed) is predictable with the model (S8 Appendix).

**Table 4:** Median (IQR) of the absolute percentage deviation of the predicted-observed-ratio (APDPOR*) in the different percentile groups of the total ED resource consumption (ln-transformed).

	Model 1		Model 2		Model 3		Model 4	
1. Decile	11.9	(4.6, 26.4)	11.7	(4.2, 26.8)	11.9	(4.8, 26.5)	11.8	(4.5, 26.3)
2. Decile	17.4	(7.7, 25.2)	16.6	(7.8, 23.9)	16.8	(7.7, 24.7)	17.8	(8.0, 25.7)
3. Decile	11.3	(8.1, 15.1)	10.9	(7.4, 14.7)	10.9	(7.5, 14.5)	11.5	(8.5, 15.3)
4. Decile	4.0	(1.7, 8.7)	4.3	(1.9, 8.8)	3.5	(1.5, 8.6)	4.2	(1.9, 8.4)
5. Decile	4.0	(1.9, 6.7)	3.9	(1.9, 6.7)	4.4	(2.1, 6.8)	3.9	(1.8, 6.8)
6. Decile	4.9	(2.6, 8.0)	5.0	(2.5, 7.8)	5.1	(2.6, 8.3)	5.0	(2.7, 8.1)
7. Decile	6.3	(3.0, 9.6)	5.9	(2.9, 9.4)	6.2	(2.9, 9.8)	6.7	(3.3, 10.1)
8. Decile	7.7	(4.0, 11.8)	7.5	(3.8, 11.4)	7.5	(3.7, 11.7)	7.7	(4.1, 12.1)
9. Decile	7.7	(3.7, 11.4)	7.1	(3.4, 11.5)	7.3	(3.5, 11.3)	7.0	(3.2, 11.1)
10. Decile	10.9	(5.9, 15.3)	10.1	(5.4, 14.7)	9.4	(4.6, 15)	10.6	(5.8, 15.2)

*APDPOR = |100%–(predicted /observed) x 100%|.

A linear regression analysis with only triage and age group as predictor variables showed an R^2^ of 23.7%. For patients with average ED resource consumption the median deviation of the predicted values is less than 8%, while the performance is worse in the lower deciles (11.9%-17.4%) as well as in the highest decile (10.9%) (Table 4). This performance measure is similar in all four studied models.

### Practical application: ED resource score and quality assurance

As a practical application example, the change in ED resource consumption over the study period was analysed (Table 5).

**Table 5:** Relative change of different performance markers at the ED compared to the baseline year 2013.

Year	Visits	Cum. RSP	HCW*	Cum. RSP / Visit	Visits / HCW	Cum. RSP / HCW
2014	+11%	+10%	+1%	-1%	+10%	+9%
2015	+21%	+21%	+10%	+/-0%	+3%	+10%
2016	+30%	+30%	+26%	+/-0%	-1%	+4%
2017	+30%	+33%	+31%	+3%	+/-0%	+2%

* HCW including all physicians and nurses at the ED.

**Abbreviations:** Cum. RSP, cumulative total ED resource score points; HCW, health care worker.

The number of visits increased over the years, increasing by 30% compared to 2013. The increase in resource needs reflected by cumulative total ED resource score points (Cum. RSP) was similar, suggesting a uniform increase over all patient resource groups. Compared to the baseline year 2013, in 2014 and 2015, the cumulative total ED resource score points per health care worker (HCW) increased by +9% and +10%, which might indicate a higher performance in the latter years compared to the baseline year.

### Length of ED stay

As a secondary outcome the LOS in the ED in hours was studied (LOS-ED). The median LOS-ED was 3.7 h (2.2–5.7) with no difference between the validation and training set (p = 0.996).

The multivariable linear regression model to predict ln-transformed LOS-ED revealed an R^2^ in the validation sample of 0.24 (Table 6). While the chief complaint showed an effect in the same direction as the analysis modelling ED resource consumption, more acute consultations, reflected by resuscitation bay use and temperature deviations, showed a GMR<1 when predicting ln-transformed LOS-ED.

**Table 6:** Multivariable analysis to predict ln-transformed LOS-ED in hours (in the training set. The exponentiated coefficients (Coef.) correspond to the GMR.

	GMR	95% CI	p-value
**Type of admission**			
Ambulance admission	1.19	(1.17, 1.20)	<0.001
**Chief complaint group**			
Cardiovascular	1.07	(1.04, 1.09)	<0.001
Ear/Nose/Throat	0.66	(0.65, 0.68)	<0.001
Eye problem	0.46	(0.45, 0.47)	<0.001
Gastrointestinal	1.27	(1.24, 1.30)	<0.001
Genitourinary	0.97	(0.94, 1.00)	<0.001
Musculoskeletal	1.00	(base)	
Neurological	1.28	(1.26, 1.31)	<0.001
Respiratory	1.10	(1.06, 1.13)	<0.001
Trauma	0.99	(0.97, 1.01)	<0.001
Other	1.02	(1.00, 1.05)	<0.001
**Acuity**			
Temperature (<35.0°C or >38.5°C)	0.78	(0.72, 0.83)	<0.001
Resuscitation bay	0.79	(0.77, 0.80)	<0.001
**Drug intake**			
On any antiepileptic	1.12	(1.10, 1.15)	<0.001
On any antihypertensive	1.13	(1.11, 1.14)	<0.001
On any antithrombotic	1.15	(1.13, 1.16)	<0.001
On any opioids	1.16	(1.14, 1.18)	<0.001
On any psycholeptic	1.11	(1.10, 1.13)	<0.001
**Comorbidity**			
Cerebrovascular disease	0.85	(0.83, 0.87)	<0.001
Dementia	1.11	(1.07, 1.15)	<0.001
Liver disease	1.22	(1.19, 1.25)	<0.001
Malignancy	1.17	(1.15, 1.19)	<0.001

R^2^ in the validation sample was 0.22.

**Abbreviations:** CI, Confidence interval; GMR, Geometric mean ratio.

## Discussion

In this retrospective analysis of a large Swiss interdisciplinary ED, the distribution of an ED patient’s consumption of resources was quantified, and predictors of total ED resource utilization were determined. Furthermore, this study developed and validated a novel scoring system for resource utilization for patients presenting to the ED that takes data acquired at the very early stages of patient care into consideration, providing better resource prediction than the use of a triage tool alone.

### Distribution of ED resource utilization

The distribution of ED resources between physician and imaging as well as laboratory was comparable to international findings [10, 11]. Resource contribution varied significantly according to triage level. The contribution of physician resources was highest for low-acuity patients, and decreased gradually with rising urgency, also comparable to international results [10, 11]. The contribution of ancillary services (laboratory work, imaging studies) showed a reverse result, with lower contribution to total ED resources in low-acuity patients and higher contribution in high-acuity patients. This finding has several implications for cost-containment and adaptive organizational measures, e.g. referral of low-acuity patients in a separate less-resource intense area of the ED and optimizing laboratory and imaging resources for high-acuity patients.

### Predicting the total ED resource consumption

Our predictors identified in univariable analysis correspond well to published findings: arrival by ambulance [40], chief complaint group [13], resuscitation bay use, deviation of vital signs [32], and triage level [6, 7, 9–11, 16].

Chief complaint has a large influence on resource utilization, with neurologic complaints showing the biggest impact, probably due to the large amount of diagnostic studies and extensive physician-patient resource needs, comparable to international findings [13], followed by cardiovascular, respiratory, gastrointestinal, and trauma. Ear-nose-throat (ENT) and ophthalmologic complaints were associated with less resource utilization. This may be explained by the fact, that these patients usually require less extensive laboratory and imaging work-up, however, there might be an underrepresentation of specialized procedural codes in the selection of the *Tarmed* catalogue. A similar finding was reported in a paediatric ENT population [41].

Regarding chronic patient conditions and contextual factors, we found an association of increasing age with resource consumption that is well described in the literature [8–10, 12, 14]. However, in our multivariable linear regression model, this difference was almost negligible. This emphasizes the important fact, that resource consumption is not associated with age *per se*, but rather with the accompanying relevant multiple comorbidities and polypharmacy, as well as cognitive or functional decline, leading to higher clinical complexity, e.g. liver disease [9].

Drug intake of any antithrombotic, antihypertensive or any opioid medication increased the geometric mean of total resource consumption about 18%, comparable to previous publications. An emergency ward setting in a tertiary hospital in Sweden reported cardiovascular medications and antithrombotic agents among the top three common drugs causing or contributing to admission [42]. Individuals with high-risk prescription opioid use are known to have significantly higher healthcare costs and utilization than their counterparts [43], and we recently demonstrated multi-substance users need significantly more ED resources than age-matched controls [44].

Similar to our predictors of resource utilization, data derived from the United States National Hospital Ambulatory Medical Care Survey also demonstrated age, triage level, arrival mode, and certain comorbidities (cerebrovascular disease, dementia) to be predictive of the eventual use of advanced diagnostic imaging in the ED [45].

With the multivariable model using easily available acute patient presentation factors as well as markers of chronic patient condition, 54% of the variance of resource utilization could be explained by the variables available at the early stages of patient presentation. These findings were verified in multiple internal sensitive analyses using different models. The identified model performed much better than a linear regression analysis with only triage and age group, two well-known predictors of resource utilization, with much poorer prediction (R^2^ = 0.24).

Furthermore, we found a high correlation of actual resource utilization with cost. However, due to very different national healthcare and billing systems [46], the results from this Swiss ER setting cannot simply be generalized to other countries, and further international studies are needed to determine, if a pure cost analysis (were data usually is more easily available) sufficiently reflects the actual resource utilization of an ED patient.

### Predicting length of ED stay

Resource consumption is difficult to measure. Some might argue that the Swiss *Tarmed* codes do not validly reflect resource consumption as a “resource measure” is already assigned to each process. Thus, we additionally evaluated the variable LOS-ED as an outcome parameter. At first glance, this might be a valid outcome variable to reflect ED resource consumption. Chief complaint showed the same direction of effect as the analysis modelling ED resource consumption, undermining the robustness of the parameter. However, a multitude of factors affect patients’ LOS-ED [13], i.e. simulation-based training for sedation procedures [47], or the use of ED observation units [48]. Furthermore, parallel and high-priority work-up of the patient is not at all reflected by LOS. High-acuity patients, treated in the resuscitation bay, are usually rapidly transferred to definite care (operating theatre, intensive/intermediate care unit), thus having a short LOS-ED, but are very resource intensive. Therefore, LOS-ED is not a suitable measure to reflect resource consumption.

### Development and validation of a scoring system

Using the variables derived in the multivariable regression, we developed and validated a novel scoring system for total ED resource consumption, taking initial information at patient presentation and the patient profile into account, explaining more than half of the variation in total ED resource consumption. These insights are crucial especially in times of resource shortages, not only for ED physicians and managers but hospital administrators and economics as well. The resource score, when applied at time of ED triage has the potential to better identify areas and special patient groups with high resource demands at an early stage.

Analogous to the TISS in the intensive care setting this score can be used be reflect resource consumption in the ED. Whereas the TISS is composed of tasks and chores actually conducted this resource score predicts ED resource consumption from the initial patient presentation, and thus it can be used to guide patient management in the ED beyond triage-category alone, i.e. assisting patient-flow by locating the patient in a more or less resource intensive area of the ED, improving medical decision-making, and providing efficient and sustainable health care. Furthermore, instead of simply presenting the volume of ED patients, it might be valuable in research as a standardised benchmarking tool to describe the service-performance of an ED and providing resource-adjusted inter-institutional cost and performance comparisons. Analogous to the TISS, which is used not only for performance measurement but also reimbursement, this score can provide hospital administrations with valuable information regarding cost generation, appropriate invoicing of emergency services provided, as well as workforce planning. Additional international multicentre studies are needed to determine how the application of such a scoring system can optimize ED resource allocation and consumption, thus providing optimal sustainable emergency care, quality assurance, and improvement.

### External validation

External validation of the suggested scoring system is the next step necessary for investigating generalizability. The predictors identified in univariable analysis correspond well to published findings also in less resource-intense settings (i.e. Lebanon) [10], which underlines a possible generalizability. Furthermore, as the score uses clinical predictors whose data collection can easily be integrated into the daily routine, e.g. triage process, it can be used in ED settings that do not collect such granular resource consumption data. The verification of external validity requires further prospective multicentre and international studies due to different intergovernmental health care and billing systems and patient populations. For example, our ED does not work under a 4-hour rule, transferring patients who need longer diagnostic work-up to an observation/short-stay unit, but takes care of the whole treatment process. Additionally, health spending varies significantly among different countries, and Switzerland is near the top of the range [46].

### Study limitations and strengths

The interpretation of our results warrants some caveats. First, data are derived from a single level one trauma and adult tertiary care referral centre, albeit one of the largest in Switzerland, making results less generalizable. Furthermore, this ED includes a large neurological referral and stroke center, a patient group prone to using a lot of resources, adding a selection bias. Next, in our definition of utilized resources we only include the direct medical resources actually documented in the ED by physician and nursing staff, as well as ancillary services, thus not considering overhead resources (i.e. infrastructure, maintenance, and hospital security). However, we have detailed records of our resource documentation, our staff is regularly trained in the documentation process, and controlling assures completeness of the documentation and circumvents variability in physician documentation practice. We focussed only on the ED process and did not include hospitalization-related resources/costs. This may lead to a partial underrepresentation of resources utilized in the third of the total patient population that required hospitalization, as some diagnostic or therapeutic measures may have been postponed. Moreover, comorbidities and medications were not standardized and automatically collected, but derived by full text parsing. Nonetheless, this was validated against manual coding. Besides that, due to a different documentation and billing system we had to exclude patients seen solely by the psychiatrist, resulting in possible selection bias, as psychiatric comorbidity may be one reason for excessive physician resource utilization [49]. Finally, whereas we recently found that resource utilization is in large part dependent on the physicians’ ratings of case difficulty (i.e. their situational level of uncertainty, familiarity and perceived difficulty), we did not include these variables in our study, which focusses on data easily available early in the patient ED presentation [50].

## Conclusions

As ED visits and health care costs are increasing globally, it is of paramount importance to understand the components of ED resource utilization, particular in times of resource scarcity. In this large retrospective study at an interdisciplinary ED, the distribution of ED resource utilization was illustrated. The novel ED resource score developed has manifold potential uses, such as an instrument i) that allows leaders in emergency care to evaluate resource decisions and to estimate effects of organizational changes, ii) to calculate benchmarks of an ED in research and process optimisation, iii) to identify resource-intensive patients more rapidly and comprehensively than triage-category alone, iv) to develop cost-containment and quality-improvement measures, and iv) that represents an easy and fast internal billing system analogous to the TISS in the intensive care unit.

## Supporting information

S1 Appendix The patient management process in our ED. (DOCX)

S2 Appendix Data extraction plan and definition of the variables. (DOCX)

S3 Appendix Validation of the diagnosis and drug parser based on agreement with 500 manually coded ED reports. (DOCX)

S4 Appendix Transformation scheme of the chief complaints by Aronsky et al. [31] to the different chief complaint main groups. (DOCX)

S5 Appendix Flowchart of the study. (DOCX)

S6 Appendix Baseline characteristics according to the type of set (Training set: n = 82,388 and validation set: n = 82,341). (DOCX)

S7 Appendix Distribution of ED resource consumption according to type of set. The median with IQR (whiskers) is shown.
(DOCX)

S8 Appendix Model fitting, predictive accuracy, and explained variance of the four different models (Model 1: Final model, Model 2: Triage instead of vital parameters, Model 3: Interaction term between trauma complaint and resuscitation room use, Model 4: Exclusion of revisits. (DOCX)
