# Supplementary material for: The development and validation of a resource consumption score of an emergency department consultation
Source: PLoS One. 2021 Feb 19;16(2):e0247244. doi: 10.1371/journal.pone.0247244 (PMC7894944; doi:10.1371/journal.pone.0247244)
Supplement: S1 Appendix — (DOCX) [file pone.0247244.s001.docx]

### S1 Appendix. The patient management process in our ED

The patient management consists of three principal components: the administration, the triage, and the clinical management process.

For administration purposes, for each consultation three identification numbers are assigned. 1. A *consultation identification number (COIN)* is linked to the clinical management process including the physician’s documentation in the clinical database. 2. A *case identification number (CID)* that is linked to the administrative data of a case. Usually there is only one COIN assigned to a CID. In rare cases, more than one COINs share the same CID, i.e. in the event of a very early revisit such as a few hours later with the same chief complain to avoid unnecessary administrative workload. However, a new medical report is created for each consultation. 3. Each patient gets a unique *patient identification number* to identify a revisit of a patient. After registration of the patient at the end of the first part of the administration process, the triage process is carried out by specially trained triage nurses using the Swiss Emergency Triage Scale (29), a triage scale similar to the Manchester Triage System (30). The triage assessment includes the assignment of the patient’s chief complaint to a triage group similar to Aronsky et al. (27), the measurement of the vital signs, and determination of the triage category from 1: highly acute to 5: non-urgent. For highly acute emergencies, the triage and first part of the administration information is documented later in the process after the acute treatment. In the clinical management phase, the physician in charge of the patient documents the patient’s history, a comprehensive diagnose list including acute and chronic diseases, clinical findings, medication intake as well the course and the discharge procedure in full text in our computerised clinical database. Additionally, the type of admission and discharge are selected from a dropdown menu.

After the clinical work-up and discharge of a patient, each staff member (nurses, physicians as well as laboratory and radiology staff) documents the procedures performed for that patient with different procedural codes from the *TARMED Suisse catalogue* (26) and store them in the administrative database (OpenText Suite for SAP® Solutions, OpenText Corporation, Waterloo, Canada) as the second phase of the administration process.

Our interdisciplinary ED is a level 1 trauma centre. All sorts of patients are treated in our ED apart from children younger than 16 years and females with gynaecological problems, as non-adults and gynaecological patients are usually treated in separate, special EDs nearby.
