# Supplementary material for: The development and validation of a resource consumption score of an emergency department consultation
Source: PLoS One. 2021 Feb 19;16(2):e0247244. doi: 10.1371/journal.pone.0247244 (PMC7894944; doi:10.1371/journal.pone.0247244)
Supplement: S2 Appendix — (DOCX) [file pone.0247244.s002.docx]

### S2 Appendix. Data extraction plan and definition of the variables.

| **Potential predictor** | **Description** | **Extraction procedure** |
| --- | --- | --- |
| **Acute patient condition** |  |  |
| Type of admission | Documented ambulance admission | Binary (1: yes, 0: no), clinical computerized database |
| Chief complaint group | Generated from the routinely documented triage group similar (27) to the following categories: 1: Cardiovascular complaint, 2: Ear/Nose/Throat complaint, 3: Eye complaint, 4: Gastrointestinal complaint, 5: Genitourinary complaint, 6: Musculoskeletal complaint, 7: Respiratory complaint, 8: Neurological complaint, 9: Trauma, 10: Other complaint; see Appendix 4 for the transformation scheme | Categorical, clinical computerized database |
| Resuscitation bay use | Use of the resuscitation bay (our ED has three of them) | Binary (1: yes, 0: no), clinical computerized database |
| Triage | Triage scale. 1: Life-threatening 2: High urgent, 3: Urgent, 4: Semi-urgent, 5: Non-urgent, 6: Missing | Categorical, clinical computerized database |
| Documented vital  deviations |  |  |
| Heart rate  (<50/min or >110/min) | Based on the first documented vital signs in the emergency report or database performed by the physician, the nurse, or automatically by monitoring. If no documentation was performed, the value assigned was "no". | Binary (1: yes, 0: no), clinical computerized database |
| Level of  consciousness  (GCS <15) |  |  |
| Oxygen saturation  (<90%) |  |  |
| Respiratory rate  (<8/min or >25/min) |  |  |
| Systolic blood  pressure (<90mmHg) |  |  |
| Temperature  (<35.0°C or >38.5°C) |  |  |
|  |  |  |

| **Contextual factors** |  |  |
| --- | --- | --- |
| Season of the year | 1: Winter (21.12.-20.03.), 2: Spring (21.01.-20.06.), 3: Summer (21.06-21.09.), 4: Fall (22.09.-20.12) | Date and time is routinely stored on arrival in the administrative database |
| Saturday or Sunday  admission | Saturday or Sunday admission from 00:01-23:59 | Binary (1: yes, 0: no), calculated from date and time |
| Night-time admissions | Night-time admissions from 19:00 to 06:59 | Binary (1: yes, 0: no), calculated from date and time |
| Occupancy index | Ratio between the total number of patients in the ED and the total number of ED treatment beds, [%] | Continuous, calculated out of administrative database |
| EDWIN | Emergency department work index,  *ES* = (Σ *n_i_* x *t_i_* ) / [*N_a_* x (*B_T_* - *B_A_*) ], where *n_i_* = number of patients in the ED in triage category *i*, *t_i_* = triage category, *N_a_* = number of attending physicians on duty, *B_T_* = the number of ED treatment beds  *B_A_* = total number of admitted patients in the ED.  0: 0-1.5, active, 1: 1.5-2.0, very busy, 2: >2, overcrowded | Categorical, calculated out of administrative database |
| **Chronic patient condition** |  |  |
| Age group | Age in years is grouped in the following categories: 1: 18-24, 2: 25-44, 3: 45-64, 4: 65-84, 5: ≥85 | Categorical, administrative database |
| Sex | Documented sex, 0: Female, 1: Male | Categorical, clinical computerized database |
| Comorbidities |  |  |
| Cerebrovascular disease | Obtained through full text parsing of the comprehensive diagnosis list. For validation of the parsing process against manually coding, see Appendix 3 | Binary (1: yes, 0: no), automatically through full text analysis of the diagnosis list in the clinical computerized database |
| Chronic kidney disease |  |  |
| COPD |  |  |
| Coronary vessel disease |  |  |
| Dementia |  |  |
| Diabetes |  |  |
| Liver disease |  |  |
| Malignancy |  |  |
| Peripheral artery disease |  |  |
| Medication intake |  |  |
| On any antidiab. (A10) | Obtained through full text parsing in the medication lists and linking the brand names to the active class to the ATC code. For validation of the parsing process against manually coding, see Appendix 3 | Binary (1: yes, 0: no), automatically through full text analysis of the medication lists in the clinical computerized database |
| On any antiepileptic (N03) |  |  |
| On any antihypertensive (C02, C04-C09) |  |  |
| On any antithrom. (B01) |  |  |
| On any diuretic (C03) |  |  |
| On any opioids (N02A) |  |  |
| On any psycholeptic (N05) |  |  |
| **Resource groups** |  |  |
| Total ED costs |  | All resource variables were obtained from the administrative database |
| Length of ED stay (hours) |  |  |
| Total ED res. consumption | Sum of the subgroups below |  |
| Physicians’ work | Sum of the subgroups |  |
| Admin time | The exact procedural codes can be obtained from the authors |  |
| Patient time |  |  |
| Report time |  |  |
| Other |  |  |
| Nurses’ work | Sum of the subgroups |  |
| Nurse patient time | The exact procedural codes can be obtained from the authors |  |
| Nurse other effort |  |  |
| Material expenses | All material expenses e.g. bandages |  |
| Laboratory resources | All laboratory expenses |  |
| Radiology resources | Sum of the subgroups |  |
| Ultrasound | The exact procedural codes can be obtained from the authors |  |
| X-ray |  |  |
| CT |  |  |
| MRI |  |  |

**Abbreviations:** antidiab., antidiabetic; antithromb., antithrombotic; COPD, chronic obstructive pulmonary disease; CT, computertomography; ED, emergency department; Magnetic Resonance Imaging; TIA, transient ischemic attack.
