# Supplementary material for: The development and validation of a resource consumption score of an emergency department consultation
Source: PLoS One. 2021 Feb 19;16(2):e0247244. doi: 10.1371/journal.pone.0247244 (PMC7894944; doi:10.1371/journal.pone.0247244)
Supplement: S3 Appendix — (DOCX) [file pone.0247244.s003.docx]

### S3 Appendix. Validation of the diagnosis and drug parser based on agreement with 500 manually coded ED reports.

|  | **Cerebrovascular disease** | **Chronic kidney disease** | **COPD** | **Coronary vessel disease** |
| --- | --- | --- | --- | --- |
| **Percent Agreement** | 0.996 (95% CI: 0.99, 1) | 0.998 (95% CI: 0.994, 1) | 0.998 (95% CI: 0.994, 1) | 0.996 (95% CI: 0.99, 1) |
| **Brennan and Prediger** | 0.992 (95% CI: 0.981, 1) | 0.996 (95% CI: 0.988, 1) | 0.996 (95% CI: 0.988, 1) | 0.992 (95% CI: 0.981, 1) |
| **Cohen/Conger's Kappa** | 0.975 (95% CI: 0.941, 1) | 0.922 (95% CI: 0.769, 1) | 0.959 (95% CI: 0.878, 1) | 0.965 (95% CI: 0.915, 1) |
| **Scott/Fleiss' Pi** | 0.975 (95% CI: 0.941, 1) | 0.922 (95% CI: 0.769, 1) | 0.959 (95% CI: 0.878, 1) | 0.965 (95% CI: 0.915, 1) |
| **Gwet's AC** | 0.995 (95% CI: 0.989, 1) | 0.998 (95% CI: 0.994, 1) | 0.998 (95% CI: 0.994, 1) | 0.996 (95% CI: 0.989, 1) |
| **Krippendorff's Alpha** | 0.975 (95% CI: 0.941, 1) | 0.922 (95% CI: 0.77, 1) | 0.959 (95% CI: 0.879, 1) | 0.965 (95% CI: 0.915, 1) |
|  | **Dementia** | **Diabetes** | **Liver disease** | **Malignancy** |
| **Percent Agreement** | 0.996 (95% CI: 0.99, 1) | 1 (95% CI: 1, 1) | 0.998 (95% CI: 0.994, 1) | 0.996 (95% CI: 0.99, 1) |
| **Brennan and Prediger** | 0.992 (95% CI: 0.981, 1) | 1 (95% CI: 1, 1) | 0.996 (95% CI: 0.988, 1) | 0.992 (95% CI: 0.981, 1) |
| **Cohen/Conger's Kappa** | 0.748 (95% CI: 0.41, 1) | 1 (95% CI: 1, 1) | 0.97 (95% CI: 0.912, 1) | 0.975 (95% CI: 0.941, 1) |
| **Scott/Fleiss' Pi** | 0.748 (95% CI: 0.409, 1) | 1 (95% CI: 1, 1) | 0.97 (95% CI: 0.912, 1) | 0.975 (95% CI: 0.941, 1) |
| **Gwet's AC** | 0.996 (95% CI: 0.99, 1) | 1 (95% CI: 1, 1) | 0.998 (95% CI: 0.994, 1) | 0.995 (95% CI: 0.989, 1) |
| **Krippendorff's Alpha** | 0.748 (95% CI: 0.409, 1) | 1 (95% CI: 1, 1) | 0.97 (95% CI: 0.912, 1) | 0.975 (95% CI: 0.941, 1) |
|  | **Peripheral artery disease** | **On any antidiabetic (A10)** | **On any antihypertensive (C02, C04-C09)** | **On any antiepileptic (N03)** |
| **Percent Agreement** | 0.998 (95% CI: 0.994, 1) | 1 (95% CI: 1, 1) | 0.996 (95% CI: 0.99, 1) | 1 (95% CI: 1, 1) |
| **Brennan and Prediger** | 0.996 (95% CI: 0.988, 1) | 1 (95% CI: 1, 1) | 0.992 (95% CI: 0.981, 1) | 1 (95% CI: 1, 1) |
| **Cohen/Conger's Kappa** | 0.951 (95% CI: 0.856, 1) | 1 (95% CI: 1, 1) | 0.985 (95% CI: 0.964, 1) | 1 (95% CI: 1, 1) |
| **Scott/Fleiss' Pi** | 0.951 (95% CI: 0.856, 1) | 1 (95% CI: 1, 1) | 0.985 (95% CI: 0.964, 1) | 1 (95% CI: 1, 1) |
| **Gwet's AC** | 0.998 (95% CI: 0.994, 1) | 1 (95% CI: 1, 1) | 0.995 (95% CI: 0.987, 1) | 1 (95% CI: 1, 1) |
| **Krippendorff's Alpha** | 0.951 (95% CI: 0.856, 1) | 1 (95% CI: 1, 1) | 0.985 (95% CI: 0.964, 1) | 1 (95% CI: 1, 1) |
|  | **On any antithrombotic (BO1)** | **On any diuretic (C03)** | **On any opioids (N02A)** | **On any psycholeptic (N05)** |
| **Percent Agreement** | 0.99 (95% CI: 0.981, 0.999) | 1 (95% CI: 1, 1) | 0.996 (95% CI: 0.99, 1) | 0.996 (95% CI: 0.99, 1) |
| **Brennan and Prediger** | 0.98 (95% CI: 0.963, 0.998) | 1 (95% CI: 1, 1) | 0.992 (95% CI: 0.981, 1) | 0.992 (95% CI: 0.981, 1) |
| **Cohen/Conger's Kappa** | 0.96 (95% CI: 0.925, 0.995) | 1 (95% CI: 1, 1) | 0.968 (95% CI: 0.925, 1) | 0.977 (95% CI: 0.945, 1) |
| **Scott/Fleiss' Pi** | 0.96 (95% CI: 0.925, 0.995) | 1 (95% CI: 1, 1) | 0.968 (95% CI: 0.925, 1) | 0.977 (95% CI: 0.945, 1) |
| **Gwet's AC** | 0.987 (95% CI: 0.975, 0.998) | 1 (95% CI: 1, 1) | 0.995 (95% CI: 0.989, 1) | 0.995 (95% CI: 0.988, 1) |
| **Krippendorff's Alpha** | 0.96 (95% CI: 0.925, 0.995) | 1 (95% CI: 1, 1) | 0.969 (95% CI: 0.925, 1) | 0.977 (95% CI: 0.945, 1) |
