# Supplementary material for: The development and validation of a resource consumption score of an emergency department consultation
Source: PLoS One. 2021 Feb 19;16(2):e0247244. doi: 10.1371/journal.pone.0247244 (PMC7894944; doi:10.1371/journal.pone.0247244)
Supplement: S4 Appendix — (DOCX) [file pone.0247244.s004.docx]

### S4 Appendix. Transformation scheme of the chief complaints by Aronsky et al. (27) to the different chief complaint main groups.

1. **Cardiovascular complaint:** cardiac arrest, cardio-vascular complications, chest pain, fainting/syncope.
2. **Ear/Nose/Throat:** ear/nose/throat problem with an otolaryngologist as the leading ED physician.
3. **Eye complaint:** ear/nose/throat problem with an ophthalmologist as the leading ED physician.
4. **Gastrointestinal complaint**: abdominal pain, abdominal problems, body aches (anal), haemorrhage.
5. **Genitourinary complaint:** flank pain, genitourinary problem.
6. **Musculoskeletal complaint:** back pain, body aches, leg pain, neck pain.
7. **Neurological complaint:** convulsions, seizures, dizziness, headache, neurological complaint, unconsciousness, stroke, and patients treated by a neurologist as the leading ED physician.
8. **Respiratory complaint:** respiratory problems.
9. **Trauma complaint:** bites, burns, fall, orthopaedic injury, electric shock, rape, stabbing, industrial/machinery accidents, gunshot wound.
10. **Other complaint:** allergies/medical reaction, needlestick/PEP, diabetic problems, procedure, medication refill, temperature related, weakness, foreign body, overdose (intentional), social problems, substance abuse, dental, toothache, fever, infection, skin complaint/trauma, fluid/nutrition alteration, follow-up, other
