# Supplementary material for: The development and validation of a resource consumption score of an emergency department consultation
Source: PLoS One. 2021 Feb 19;16(2):e0247244. doi: 10.1371/journal.pone.0247244 (PMC7894944; doi:10.1371/journal.pone.0247244)
Supplement: S5 Appendix — (DOCX) [file pone.0247244.s005.docx]

### S5 Appendix. Flowchart of the study

ED consultations from 01.01.2013 - 31.12.2017
**(n = 206,006)**

Records excluded **(n = 41,277)**:

- Age < 18 years (n=6,992)
- Case identification number missing or with multiple consultations (n=7,478)
- No resource database entry (n=4,977)
- Patients were seen by the psychiatrist as the leading physician (n=8,511)
- Total resources smaller than 10 tax points (n=2,662)
- No documented chief complaint (n=10,657)

ED consultations included in the study
**(n = 164,729)**
