# Supplementary material for: The development and validation of a resource consumption score of an emergency department consultation
Source: PLoS One. 2021 Feb 19;16(2):e0247244. doi: 10.1371/journal.pone.0247244 (PMC7894944; doi:10.1371/journal.pone.0247244)
Supplement: S6 Appendix — (DOCX) [file pone.0247244.s006.docx]

### S6 Appendix. Baseline characteristics according to the type of set (Training set: n=82,388 and Validation set: n=82,341). Chi-squared respectively Wilcoxon rank-sum test, as appropriate

|  | **Training set** | | **Validation set** | | **Total** |  | **p-value** |
| --- | --- | --- | --- | --- | --- | --- | --- |
| **Sociodemographic characteristics** | | |  |  |  |  |  |
| Age group, years |  |  |  |  |  |  |  |
| 18-24 | 9,287 | (11.3) | 9,263 | (11.2) | 18,550 | (11.3) |  |
| 25-44 | 25,857 | (31.4) | 25,894 | (31.4) | 51,751 | (31.4) |  |
| 45-64 | 24,103 | (29.3) | 23,993 | (29.1) | 48,096 | (29.2) |  |
| 65-84 | 19,409 | (23.6) | 19,421 | (23.6) | 38,830 | (23.6) |  |
| ≥85 | 3,732 | (4.5) | 3,770 | (4.6) | 7,502 | (4.6) | 0.974 |
| Sex |  |  |  |  |  |  |  |
| Female | 35,920 | (43.6) | 36,017 | (43.7) | 71,937 | (43.7) |  |
| Male | 46,468 | (56.4) | 46,324 | (56.3) | 92,792 | (56.3) | 0.559 |
| **Type of admission** |  |  |  |  |  |  |  |
| Ambulance admission | 12,697 | (15.4) | 12,909 | (15.7) | 25,606 | (15.5) | 0.136 |
| **Chief complaint group** |  |  |  |  |  |  |  |
| Cardiovascular | 6,735 | (8.2) | 6,692 | (8.1) | 13,427 | (8.2) |  |
| Ear/Nose/Throat | 6,859 | (8.3) | 6,878 | (8.4) | 13,737 | (8.3) |  |
| Eye problem | 5,120 | (6.2) | 5,001 | (6.1) | 10,121 | (6.1) |  |
| Gastrointestinal | 7,289 | (8.8) | 7,406 | (9.0) | 14,695 | (8.9) |  |
| Genitourinary | 3,011 | (3.7) | 2,932 | (3.6) | 5,943 | (3.6) |  |
| Musculoskeletal | 5,196 | (6.3) | 5,161 | (6.3) | 10,357 | (6.3) |  |
| Neurological | 13,496 | (16.4) | 13,388 | (16.3) | 26,884 | (16.3) |  |
| Respiratory | 3,303 | (4.0) | 3,287 | (4.0) | 6,590 | (4.0) |  |
| Trauma | 13,623 | (16.5) | 13,862 | (16.8) | 27,485 | (16.7) |  |
| Other | 17,756 | (21.6) | 17,734 | (21.5) | 35,490 | (21.5) | 0.718 |
| **Resuscitation bay use** | 4,564 | (5.5) | 4,597 | (5.6) | 9,161 | (5.6) | 0.702 |

| **Triage** |  |  |  |  |  |  |  |
| --- | --- | --- | --- | --- | --- | --- | --- |
| Life-threatening | 6,659 | (8.1) | 6,697 | (8.1) | 13,356 | (8.1) |  |
| High urgent | 20,139 | (24.4) | 19,956 | (24.2) | 40,095 | (24.3) |  |
| Urgent | 49,911 | (60.6) | 50,074 | (60.8) | 99,985 | (60.7) |  |
| Semi-urgent | 3,993 | (4.8) | 3,923 | (4.8) | 7,916 | (4.8) |  |
| Non-urgent | 538 | (0.7) | 518 | (0.6) | 1,056 | (0.6) |  |
| Missing | 1,148 | (1.4) | 1,173 | (1.4) | 2,321 | (1.4) | 0.782 |
| **Documented vital deviations** |  |  |  |  |  |  |  |
| Heart rate (<50/min or >110/min) | 702 | (0.9) | 665 | (0.8) | 1,367 | (0.8) | 0.320 |
| Level of conscious (GCS <15) | 7,925 | (9.6) | 7,836 | (9.5) | 15,761 | (9.6) | 0.479 |
| Oxygen saturation (<90%) | 1,235 | (1.5) | 1,251 | (1.5) | 2,486 | (1.5) | 0.736 |
| Respiratory rate (<8/min or >25/min) | 2,741 | (3.3) | 2,851 | (3.5) | 5,592 | (3.4) | 0.129 |
| Systolic blood pressure (<100mmHg) | 2,569 | (3.1) | 2,588 | (3.1) | 5,157 | (3.1) | 0.772 |
| Temperature (<35.0°C or >38.5°C) | 336 | (0.4) | 321 | (0.4) | 657 | (0.4) | 0.563 |
| **Comorbidities** |  |  |  |  |  |  |  |
| Cerebrovascular disease | 6,074 | (7.4) | 5,939 | (7.2) | 12,013 | (7.3) | 0.212 |
| Chronic kidney disease | 1,806 | (2.2) | 1,851 | (2.2) | 3,657 | (2.2) | 0.441 |
| COPD | 2,313 | (2.8) | 2,278 | (2.8) | 4,591 | (2.8) | 0.614 |
| Coronary vessel disease | 6,919 | (8.4) | 6,884 | (8.4) | 13,803 | (8.4) | 0.782 |
| Dementia | 1,502 | (1.8) | 1,511 | (1.8) | 3,013 | (1.8) | 0.856 |
| Diabetes | 6,947 | (8.4) | 6,737 | (8.2) | 13,684 | (8.3) | 0.066 |
| Liver disease | 2,493 | (3.0) | 2,422 | (2.9) | 4,915 | (3.0) | 0.314 |
| Malignancy | 7,731 | (9.4) | 7,509 | (9.1) | 15,240 | (9.3) | 0.064 |
| Peripheral artery disease | 1,588 | (1.9) | 1,656 | (2.0) | 3,244 | (2.0) | 0.222 |
| **Drug intake** |  |  |  |  |  |  |  |
| On any antidiabetic | 4,994 | (6.1) | 4,887 | (5.9) | 9,881 | (6.0) | 0.280 |
| On any diuretic | 7,399 | (9.0) | 7,319 | (8.9) | 14,718 | (8.9) | 0.513 |
| On any antiepileptic | 5,155 | (6.3) | 5,060 | (6.1) | 10,215 | (6.2) | 0.347 |
| On any antihypertensive | 18,322 | (22.2) | 18,111 | (22.0) | 36,433 | (22.1) | 0.234 |
| On any antithrombotic | 18,512 | (22.5) | 18,366 | (22.3) | 36,878 | (22.4) | 0.423 |
| On any opioids | 6,624 | (8.0) | 6,476 | (7.9) | 13,100 | (8.0) | 0.189 |
| On any psycholeptic | 8,249 | (10.0) | 8,120 | (9.9) | 16,369 | (9.9) | 0.306 |
| **Contextual factors** |  |  |  |  |  |  |  |
| Season |  |  |  |  |  |  |  |
| Winter | 19,933 | (24.2) | 19,792 | (24.0) | 39,725 | (24.1) |  |
| Spring | 20,941 | (25.4) | 20,840 | (25.3) | 41,781 | (25.4) |  |
| Summer | 21,378 | (25.9) | 21,560 | (26.2) | 42,938 | (26.1) |  |
| Fall | 20,136 | (24.4) | 20,149 | (24.5) | 40,285 | (24.5) | 0.681 |
| Night-time admissions (19:00 – 06:59) | 26,384 | (32.0) | 26,146 | (31.8) | 52,530 | (31.9) | 0.238 |
| Saturday or Sunday admission (00:00-23:59) | 24,437 | (29.7) | 24,376 | (29.6) | 48,813 | (29.6) | 0.800 |
| Occupancy index [%], median (IQR) | 59 | (44 - 73.5) | 58.8 | (44.1 - 73.5) | 58.8 | (44.1 - 73.5) | 0.601 |
| EDWIN score |  |  |  |  |  |  |  |
| 0-1.5, active | 74,720 | (90.7) | 74,630 | (90.6) | 149,350 | (90.7) |  |
| 1.5-2.0, very busy | 7,427 | (9.0) | 7,486 | (9.1) | 14,913 | (9.1) |  |
| >2, overcrowded | 241 | (0.3) | 225 | (0.3) | 466 | (0.3) | 0.662 |

**Abbreviations:** IQR, interquartile range
