# Supplementary material for: The development and validation of a resource consumption score of an emergency department consultation
Source: PLoS One. 2021 Feb 19;16(2):e0247244. doi: 10.1371/journal.pone.0247244 (PMC7894944; doi:10.1371/journal.pone.0247244)
Supplement: S7 Appendix — The median with IQR (whiskers) is shown. (DOCX) [file pone.0247244.s007.docx]

### S7 Appendix. Distribution of ED resource consumption according to type of set. The median with IQR (whiskers) is shown.

No significant differences were found in any resource group (p>0.1).
