# Supplementary material for: The development and validation of a resource consumption score of an emergency department consultation
Source: PLoS One. 2021 Feb 19;16(2):e0247244. doi: 10.1371/journal.pone.0247244 (PMC7894944; doi:10.1371/journal.pone.0247244)
Supplement: S8 Appendix — (DOCX) [file pone.0247244.s008.docx]

### S8 Appendix. Model fitting, predictive accuracy, and explained variance of the four different models (Model 1: Final model, Model 2: triage instead of vital parameters, Model 3: interaction term between trauma complaint and resuscitation room use, Model 4: exclusion of revisits.

| **Model** | **Model fitting*** | |  | **Predictive accuracy^#^** | | | | |  | **Explained variance^#^** |
| --- | --- | --- | --- | --- | --- | --- | --- | --- | --- | --- |
|  | AIC | BIC |  | MSPE | Bias | MRSE | | MAPE |  | R^2^ |
| 1 | 191215 | 191476 |  | 914339 | -195 | | 540 | 481 |  | 0.54 |
| 2 | 187200 | 187452 |  | 866736 | -189 | | 502 | 468 |  | 0.57 |
| 3 | 188863 | 189142 |  | 838834 | -189 | | 491 | 459 |  | 0.55 |
| 4 | 124057 | 124305 |  | 1108380 | -208 | | 596 | 512 |  | 0.56 |

*Development sample, **^#^** Validation sample

**Abbreviations:** AIC, Akaike information criterion; BIC, Bayesian information criterion; MAPE, mean absolute prediction error; MRSE, mean relative squared error; MSPE, mean squared error prediction.

MSPE = 1/n x [ Σ (predicted_k_ - observed_k_)^2^ ], Bias = 1/n x [ Σ (predicted_k_ - observed_k_)],

MRSE = 1/n x [ Σ (predicted_k_ - observed_k_)^2^ / (observed_k_ +1)], MAPE = 1/n x [ Σ |predicted_k_ - observed_k_|]
